# Evaluating the Role of Exogenously Applied Ascorbic Acid in Rescuing Soybean Plant Health in The Presence of Pathogen-Induced Oxidative Stress

**DOI:** 10.3390/pathogens11101117

**Published:** 2022-09-28

**Authors:** Afsana Noor, Christopher R. Little

**Affiliations:** Department of Plant Pathology, Kansas State University, Manhattan, KS 66506, USA

**Keywords:** *Macrophomina phaseolina*, charcoal rot, ascorbic acid, ROS scavenger, H_2_O_2_

## Abstract

Charcoal rot, caused by the soilborne hemibiotrophic fungus *Macrophomina phaseolina*, is a prevalent and economically significant plant disease. It is hypothesized that *M. phaseolina* induces oxidative stress-mediated senescence in plants. Infection by *M. phaseolina* results in the host’s accumulation of reactive oxygen species (ROS) that contribute toward basal defense. However, the production of ROS could also lead to cellular damage and senescence in host tissue. This study aimed to determine if ascorbic acid, a ROS scavenging molecule, could quench *M. phaseolina*-induced hydrogen peroxide (H_2_O_2_) generation in a soybean-*M. phaseolina* pathosystem. In vitro sensitivity tests showed that *M. phaseolina* isolates were sensitive to L-ascorbic acid (LAA) at concentrations of 10.5 to 14.3 mM based on IC_50_ (half-maximal inhibitory concentration) data. In planta cut-stem assays demonstrated that pre-treatment with 10 mM of either LAA (reduced form) or DHAA (dehydroascorbic acid; oxidized form) significantly decreased lesion length compared to the non-pretreated control and post-treatments with both ascorbic acid forms after *M. phaseolina* inoculation. Further, H_2_O_2_ quantification from ascorbic acid-pretreated tissue followed by *M. phaseolina* inoculation showed significantly less accumulation of H_2_O_2_ than the inoculated control or the mock-inoculated control. This result demonstrated that *M. phaseolina* not only induced H_2_O_2_ after host infection but also increased ROS-mediated senescence. This study shows the potential of ascorbic acid, an effective ROS scavenger, to limit ROS-mediated senescence associated with *M. phaseolina* infection.

## 1. Introduction

Charcoal rot of soybean (*Glycine max* (L.) Merr.) is caused by the ubiquitous soilborne fungus *Macrophomina phaseolina* (Tassi) Goid. *M. phaseolina* infects a wide range of plant hosts and is distributed worldwide [1,2,3,4,5]. Under favorable environmental conditions, host infection begins with the germination of microscelerotia in the soil near plant roots. Then, microsclerotial hyphae penetrate the soybean plant through the root and grow intercellularly into the vascular region. Fungal colonization ultimately damages plant tissue by plugging xylem vessels and producing phytotoxins [6,7,8,9]. When no conspicuous disease symptoms are expressed in the plant, the fungus remains biotrophic and latent. A histopathological study using a resistant and susceptible soybean cultivar revealed that *M. phaseolina* requires a nine-day incubation or biotrophic phase in a susceptible cultivar to grow intercellularly and colonize the stele tissue. In contrast, the resistant cultivar restricted infection and disease development [10]. However, with the onset of an environmental stressor, such as drought or high temperature, high plant populations, or nutritional deficiency during the post-flowering stages, *M. phaseolina* may induce necrotrophy [8,11]. In the necrotrophic phase, *M. phaseolina* causes disease symptoms associated with wilting as the pathogen blocks the vascular bundles, induces the production of plant degradative enzymes, and causes phytotoxin-mediated necrosis [1,8,10,12]. Therefore, *M. phaseolina* functions as a hemibiotroph by taking advantage of both the biotrophic phase needed for its initial infection and development and the progression of the necrotrophic phase, which is required for pathogen survival [10].

Plants possess two layers of defenses against microorganisms, basal and specific. Specific defense is mediated by resistance (R) genes. It produces a hypersensitive response (HR) in incompatible interactions, whereas basal defense recognizes microbe-associated molecular patterns (MAMPs), which triggers MAMP-triggered immunity (MTI) [13,14,15,16]. Biotrophic pathogens depend on living hosts for nutrition and proliferation. At the same time, their growth is restricted once the host R gene recognizes the biotrophic pathogen-secreted effector, and the host induces HR-mediated cell death. However, pathogens with necrotrophic phases, such as *M. phaseolina*, take advantage of this R gene-mediated necrotrophic effector recognition and HR-mediated built-in cell senescence as their growth, nutrition, infection, and further proliferation initiate with the R gene-mediated defense responses and host senescence. This host defense mechanism benefits pathogens with necrotrophic phases to become more infective and ultimately increases host susceptibility [17,18].

Reactive oxygen species (ROS) are generated as defense signals in plants in response to abiotic and biotic stresses [12,19,20]. ROS species include H_2_O_2_ (hydrogen peroxide), OH^•^ (hydroxyl radical), and ^•^O_2_^–^ (superoxide radical) [21]. Of these, H_2_O_2_ is long-lived and is a REDOX (reduction-oxidation) metabolite that acts as a signaling molecule or an inducer of oxidative damage depending on its concentration [22,23]. Low and balanced levels of H_2_O_2_ are known to be involved in diverse signaling processes in the plant, including stress tolerance, control of enzymatic and nonenzymatic antioxidative defense systems (the plant has a built-in antioxidative defense system to mask against oxidative damage caused by these free toxic and reactive oxygen intermediates), stomatal control, cell cycle, growth regulation, and photosynthesis [22,23,24]. However, imbalanced or excessive H_2_O_2_ induces toxicity and damage to biomolecules, which leads to cellular injury and cell death [22,23].

A previous study showed that *M. phaseolina* induced ROS generation in sorghum [12] and RNS (reactive nitrogen species) generation in jute after inoculation [20]. ROS could act as a defense signal-related response against the biotic stress conferred by the pathogen or insects and abiotic stress conferred by non-biotic entities if the balance between ROS generation and scavenging is maintained [25]. However, if excess ROS generated by abiotic and biotic stressors are not scavenged, the cellular homeostasis will be lost, and resulting malfunctions in plant metabolic activity could lead to oxidative damage-related cell senescence [26]. If *M. phaseolina* induces oxidative stress-mediated senescence in soybean, we hypothesize that ascorbic acid, a reactive oxygen scavenger of H_2_O_2_, can mitigate pathogen-mediated senescence. The specific objectives were: (i) to test the in vitro sensitivity of *M. phaseolina* isolates against inhibition by ascorbic acid; (ii) to apply ascorbic acid to wounded and inoculated soybean seedling stems in an in-planta assay to determine its ability to reduce *M. phaseolina*-associated development; and (iii) to validate the ROS (H_2_O_2_) induced by *M. phaseolina* in the soybean host using a spectrophotometric absorbance assay.

## 2. Materials and Methods

### 2.1. Soybean Genotype and Macrophomina phaseolina Isolates

**Figure 1:**
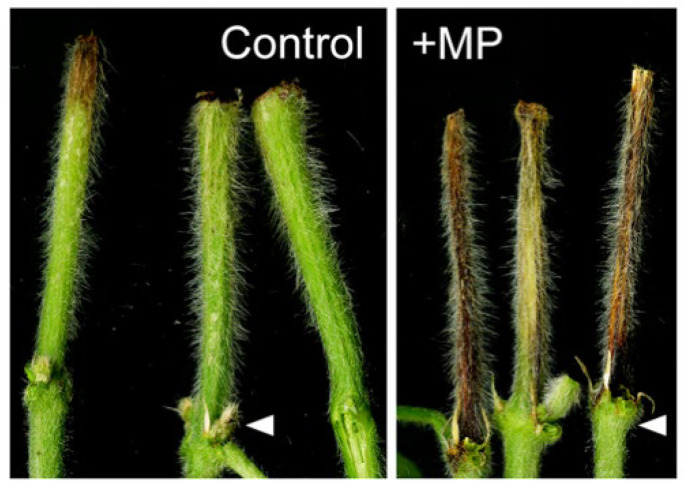
Lesion development on soybean variety ‘AG3039′ after inoculation with agar plug (1/4-strength PDA (potato dextrose agar) plug; “Control”) and *Macrophomina phaseolina* (“+MP”) using the cut-stem assay (see Section 2). White arrows indicate the unifoliate node.

The commonly grown soybean genotype AG3039 (Asgrow, Bayer Crop Science, Creve Coeur, MS, USA) was used for this experiment. Susceptibility of AG3039 to *M. phaseolina* was established using the seedling stem necrosis assay (Figure 1) [8].

Three *M. phaseolina* isolates were selected for testing in this study. These included MP110, MP154, and MP336. The isolates were obtained from soybeans grown in Rossville (in 2002), Leavenworth (in 2002), and Manhattan (Ashland Bottoms Research Farm, Kansas State University, Manhattan, KS, USA; in 2008), Kansas, respectively. MP110 and MP154 were previously characterized by Saleh et al. [27]. In addition to cultural and morphological characteristics, the rDNA-ITS region of MP336 was sequenced using the *MpKF1* and *MpKR1* primer set developed by Babu et al. [28] to confirm its identity.

### 2.2. In Vitro Sensitivity of M. phaseolina to Ascorbic Acid

**Figure 2:**
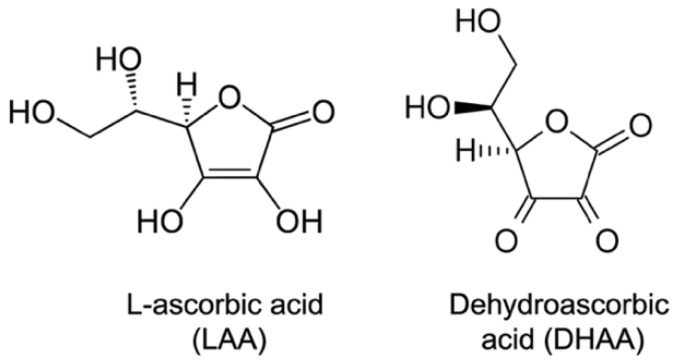
Reduced (L-ascorbic acid, LAA) and oxidized (dehydroascorbic acid, DHAA) used in this study.

For the in vitro growth test, 1/4-strength potato dextrose agar (PDA; Difco Laboratories, Detroit, MI, USA) media was amended with ten concentrations (0, 0.5, 5, 10, 15, 20, 25, 30, 40, and 50 mM) of ascorbic acid (L-ascorbic acid; Millipore Sigma, Burlington, MA, USA; Figure 2). The ascorbic acid was added once the media cooled < 55 °C after autoclaving. *M. phaseolina* isolates MP336, MP154, and MP110 were plated on the media, and growth was recorded every 24 h for 5 days. The experimental design was a RCBD (randomized complete block design) with factorial (*M. phaseolina* isolates and ascorbic acid concentrations) with three replications. In addition, IC_50_ (half-maximal inhibitory concentration) values were calculated from daily measurements using the Quest GraphTM IC_50_ calculator online tool [29]. DHAA (dehydroascorbic acid) was not tested in the in vitro sensitivity experiment.

### 2.3. Soybean Stem Necrosis Assay

These experiments were conducted in the Throckmorton Plant Sciences greenhouse facilities at Kansas State University, Manhattan, KS, USA. The experimental design was a RCBD with three replications and twelve treatments. The experiment was repeated twice.

Five soybean seeds were planted in 12.7 × 12.7 cm (5 × 5 in.) pot, and seedlings thinned to three plants per pot once they reached the cotyledonary stage (VC). The remaining seedlings were grown until the 2nd trifoliate (V2) stage.

The cut-stem inoculation method of Twizeymania et al. [8] was followed for this experiment. The apical portions of V2 soybean seedlings were cut 25 mm above the unifoliate node. Inverted two hundred microliter (200 µL) micropipette tubes containing one of the pre-treatments and treatments listed in Table 1 were placed on the cut portion of the seedling. To apply the ascorbic acid or H_2_O_2_ exogenously, a small sponge piece was inserted inside the micropipette, and it was soaked with 100 µL of the respective liquid (ascorbic acid or H_2_O_2_). Sterile agar plugs (~5 mm diameter × ~8 mm depth) were used as the negative control. Agar plugs of the same dimensions, colonized with *M. phaseolina* (isolate MP336), were placed fungal colonized-side down on the cut-stem and used as the positive control and inoculated treatments.

### 2.4. H_2_O_2_ Absorbance Assay

To show that *M. phaseolina* induces the production of the reactive oxygen species (ROS), specifically H_2_O_2_, in the soybean stem assay, mock-inoculated, inoculated, ascorbic acid pre-treated, and inoculated soybean stems were processed using the method from Veljovic-Jovanovic et al. [30] and Liu et al. [31]. Briefly, cut stems were collected at the 2nd trifoliate stage (including the top, middle, and bottom portion of the cotyledonary node to 25 mm above it) and immediately frozen in liquid nitrogen. The tissue was homogenized in 1.5 mL 1 M HClO_4_ (perchloric acid) with 100 mg polyvinylpyrrolidone (to remove phenolic compounds) (Millipore Sigma, Burlington, MA, USA). The homogenate was centrifuged at 13,000× *g* for 10 min at 4 °C. Following the method of Cheesseman [32] and Liu et al. [31], 60 mL of centrifuged stem tissue extract was mixed with 600 mL of eFOX (ferrous oxidation-xylenol orange) reagent, which contained 250 µM ferrous ammonium sulfate, 100 µM sorbitol, 100 µM xylenol orange, and 1% methanol combined in 25 mM H_2_SO_4_ (sulfuric acid), and 1% ethanol (Millipore Sigma, Burlington, MA, USA). Thirty minutes after mixing tissue extract with the eFOX reagents, spectrophotometric absorbance was measured at 550 and 800 nm using a plate reader (BioTek Synergy H1 Hybrid Multi-Mode Microplate Reader; BioTek Instruments, Inc., Winooski, VT, USA). Quantification of H_2_O_2_ was calibrated using an H_2_O_2_ standard curve.

### 2.5. Statistics

For the in vitro sensitivity assay, analysis of variance (ANOVA) was performed using the PROC GLM procedure of SAS 9.4 (SAS Institute, Cary, NC, USA). Across isolates and days post-plating (DPPL), mean separations were conducted for ascorbic acid concentration, and *M. phaseolina* isolates using LSMEANS of the SAS PROC GLM procedure. Within isolates and DPPL, the student’s *t*-test (JMP 16.2.0; SAS Institute, Inc., Cary, NC, USA) was used to compare growth on 0 mM ascorbic acid to each individual concentration of ascorbic acid, for the cut-stem assay, ANOVA was conducted using the PROC GLIMMIX procedure of SAS. Means separations for areas under the disease progress curve (AUDPC) values were performed using LSMEANS of the SAS GLIMMIX procedure. ANOVA for the H_2_O_2_ absorbance assay was carried out using the PROC GLM procedure of SAS. Means separation was performed using the Least Significant Difference (LSD) of the mean. Box plots were used for the graphical representation of AUDPC and H_2_O_2_ quenching by ascorbic acid [33].

## 3. Results

### 3.1. In Vitro Sensitivity Assay

**Figure 3:**
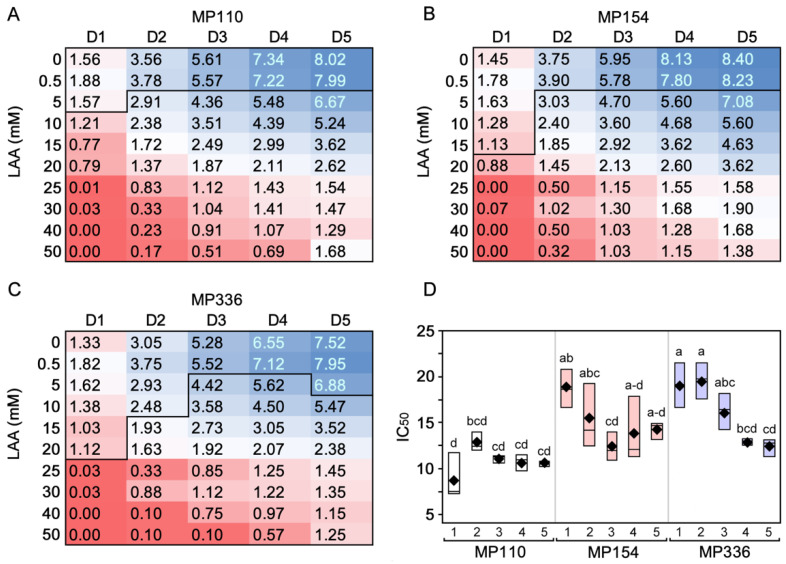
Diameters of three isolates of *Macrophomina phaseolina* (MP110, −154, and −336) grown on 0 to 50 mM L-ascorbic acid (LAA)-amended 1/4-strength PDA. Average colony growth (cm) of MP110 (**A**), MP154 (**B**), and MP336 (**C**). Values below the horizontal line differ from the “0” control at *P* < 0.05 according to a student’s *t*-test for specific days after plating. Box plots show IC_50_ (half-maximal inhibitory concentration) values (bottom right) with black diamonds representing mean values (**D**). IC_50_ values with different letters significantly differ according to Tukey’s Honestly Significantly difference test (*P* < 0.05).

Comparison of colony growth on media containing ascorbic acid with media not containing ascorbic acid (i.e., 0 mM) was significantly decreased. At 3 and 4 DPPL, all three isolates grown on 5 mM ascorbic acid showed significantly decreased growth compared to control plates. However, at 1 DPPL, MP110, MP154, and MP336 differed from control at 10 mM, 20 mM, and 25 mM, respectively. At 2 DPPL, MP110 and MP154 differed from control at 5 mM, whereas MP336 differed at 15 mM. At 5 DPPL, MP110 and MP154 differed from control at 5 mM, whereas MP336 differed at 10 mM. Overall, MP154 and MP336 exhibited a significantly higher IC_50_ than MP110 (*F* = 13.96; *P* < 0.0001) (Table 2; Figure 3).

**Figure 4:**
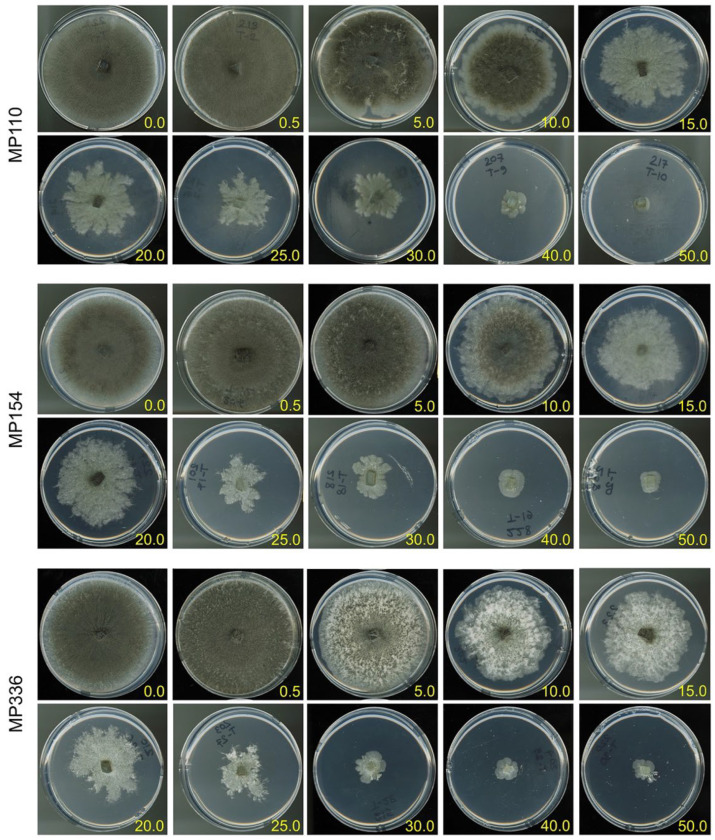
*Macrophomina phaseolina* growth morphology on varying concentrations of L-ascorbic acid. Three soybean isolates (MP110, −154, and −336) were grown on quarter-strength PDA amended with 0 to 50 mM L-ascorbic acid.

At 10 to 15 mM ascorbic acid concentrations, all *M. phaseolina* isolates exhibited reduced microsclerotia production. At the same concentrations, cultures transitioned from a circular form with entire margins to an irregularly formed colony with lobate to filiform margins (Figure 4).

Based on these results, MP336 was chosen as the test isolate for the remainder of the experiments.

### 3.2. Cut-Stem Assay

**Figure 5:**
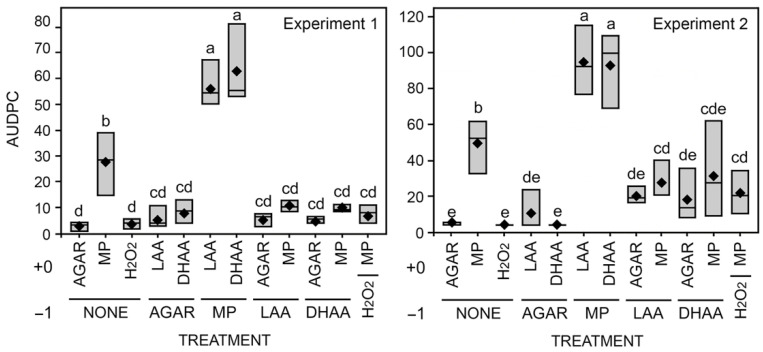
Areas under the disease progress curve (AUDPC) for lesion development in the greenhouse cut-stem assay experiments. Pre-treatments were performed one day in advance (−1) of treatments (+0), and stem lesions were monitored after +3, +5, and +7 days after inoculation. Abbreviations: DHAA = dehydroascorbic acid (oxidized form); LAA = L-ascorbic acid (reduced form); H_2_O_2_ = hydrogen peroxide; MP = *Macrophomina phaseolina* inoculation. Black diamonds represent mean values within the box plots.

**Figure 6:**
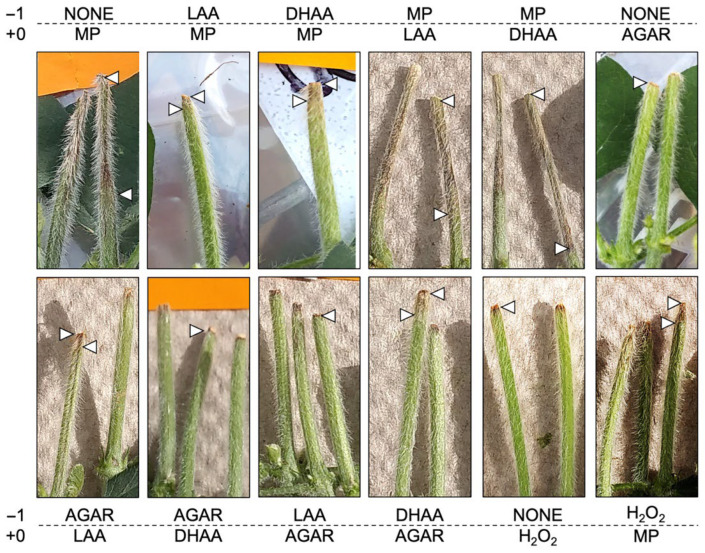
Representative soybean cut-stem pathogenicity assay lesion development after treatments were applied in this experiment. Pre-treatments (−1) were performed one day prior to treatments (+0). White arrows indicate the approximate extent of necrotic lesions in cut stems. Abbreviations: DHAA = dehydroascorbic acid (oxidized form), LAA = L-ascorbic acid (reduced form), MP = Macrophomina phaseolina.

In the cut-stem assay, *M. phaseolina*-inoculated soybean plants produced significantly longer lesions than agar alone. Furthermore, when cut stems were inoculated with *M. phaseolina* and subsequently treated with dehydroascorbic acid (DHAA) and L-ascorbic acid (LAA) (day +0), lesion length on the stem was significantly longer than the inoculated plants (Figure 5 and Figure 6). However, when cut stems were pre-treated with DHAA and LAA (day −1), lesion length was significantly reduced compared to inoculation alone and not different than agar after ascorbic acid treatment (Figure 5 and Figure 6).

### 3.3. H_2_O_2_ Absorbance Assay

**Figure 7:**
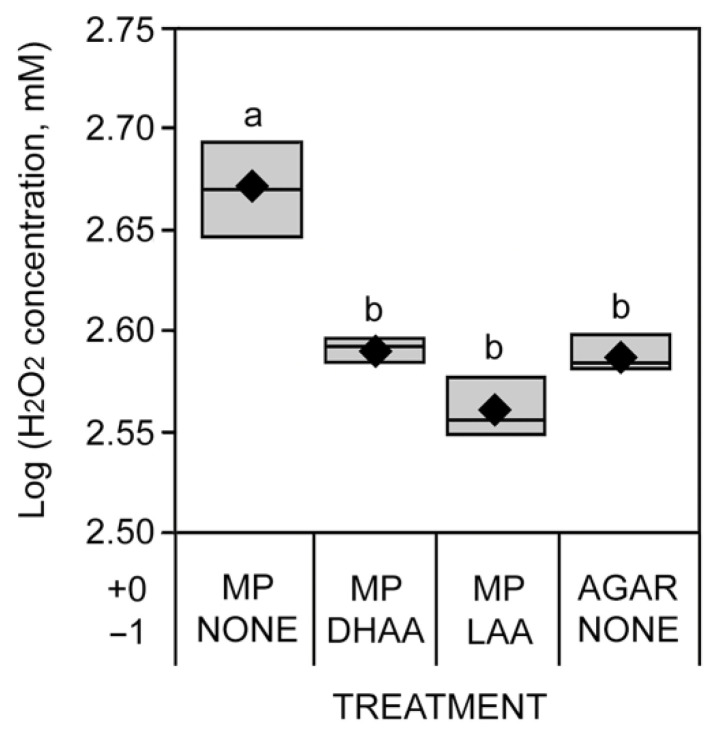
Ascorbic acid quenches pathogen-mediated H_2_O_2_ generation in the soybean cut-stem assay. Pre-treatments occurring one day before treatments are denoted by “−1” and “+0,”, respectively. Abbreviations: DHAA = dehydroascorbic acid (oxidized form), LAA = L-ascorbic acid (reduced form), MP = *Macrophomina phaseolina*. Black diamonds represent mean values within box plots.

To confirm that *M. phaseolina* induces the production of H_2_O_2_, absorbance was measured from inoculated plants in vitro (Table 3; Figure 7). *M. phaseolina* inoculation (positive control) increased H_2_O_2_ concentration from soybean stem tissue extracts compared to no pre-treatment and subsequent inoculation with agar (negative control). Likewise, pre-treatment with LAA or DHAA and subsequent inoculation with *M. phaseolina* after 1 day significantly reduced H_2_O_2_ compared to the positive control and did not differ from the negative control (Table 3; Figure 7).

## 4. Discussion

To investigate pathogen-induced ROS-mediated senescence in soybean, reduced (LAA) and oxidized (DHAA) forms of ascorbic acid were tested as ROS scavengers to determine their potential to minimize ROS-induced effects in planta. LAA was used to test in vitro sensitivity of *M. phaseolina*. In this study, *M. phaseolina* isolates showed sensitivity to LAA concentrations > 10 mM. Botanga et al. [34], using a similar in vitro assay, tested the growth of the necrotrophic fungus, *Alternaria brassicicola*, against various concentrations of ascorbic acid in minimal media. Their study demonstrated that fungal growth was inhibited at 25 mM and restricted at 10 mM. Based on the current study and Botanga et al. [34], it may be concluded that higher ascorbic acid concentrations are toxic and inhibitory to fungal growth. However, specific levels may vary between fungal species or isolates of the same species. However, the ascorbic acid-sensitive fungal metabolic targets, whether direct or indirect via pH changes, remain unclear.

Ascorbic acid has been used in plant systems to evaluate its activity in scavenging abiotic stress-mediated ROS generation [19]. For example, exogenously applied ascorbic acid actively protected lipids and proteins from drought and salt stress [35,36,37]. Ascorbate, which is a regenerative and potent antioxidant molecule [19,38,39], has shown promising ROS neutralizing capabilities along with vitamin E through direct and indirect actions (enzyme catalysis) [19,38,39]. Endogenous ascorbate has a significant role in antioxidative metabolism. Exogenous application of ascorbic acid has been found to increase endogenous ascorbic acid levels [19,40,41]. Ascorbic acid has the potential to activate antioxidant molecules, and ascorbate peroxidase (APx) is known to dismute the major ROS, H_2_O_2_, to water and oxygen [19,42,43]. Khan and Ashraf [40] assessed the role of exogenously applied ascorbic acid (0, 50, and 100 mg L^−1^) in attenuating the salt toxicity effect on two wheat (*Triticum aestivum* L.) cultivars (salt tolerant and moderately salt sensitive) when grown under normal conditions (0 mM NaCl) and a salt treated condition (150 mM NaCl). Their study revealed that foliar sprays of ascorbic acid shielded the photosynthetic machinery of both wheat cultivars against the toxic effect of the salt treatment and rescued plants from salt-induced chlorophyll-*a* content reduction. Additionally, exogenously applied ascorbic acid rescued okra (*Abelmoschus esculentus* (L.) Moench) plants from oxidative stress-associated electrolyte leakage and lipid peroxidation during drought stress conditions [44].

Basal defense in plants upon microbe recognition is initiated through ROS signaling. For example, ROS was generated in the initial, short-lived biotrophic phase of *M. phaseolina* upon recognition by both susceptible and resistant sesame (*Sesamum indicum* L.) plants. However, ROS generation increased significantly in susceptible sesame plants compared to the resistant host once the fungus switched from the biotrophic to the necrotrophic phase [45]. ROS generation is a normal plant phenomenon during any physiological or metabolic process [46,47]. Biotic and abiotic stressors induce excessive ROS, which leads to redox imbalances and oxidative stress in the plant [21,47,48]. Plants possess antioxidant machinery comprised of both enzymatic (superoxide dismutase (SOD), catalase (CAT), ascorbate peroxidase, glutathione peroxidase (GPx), glutathione-S-transferase, monodehydroascorbate reductase, and dehydroascorbate reductase) and non-enzymatic compounds (ascorbate, phenolic compounds, carotenoids, and tocopherol) to mitigate such redox imbalances [46,47]. Therefore, plants have an innate “immune” reaction against the oxidative damage caused by ROS through the secretion of antioxidants and other substances that can scavenge ROS [19]. In susceptible plants, it is possible that excess production of ROS cannot be fully quenched by host antioxidants, which results in further accumulation of ROS and oxidative damage to cells [49].

Our results showed that 10 mM of exogenously applied ascorbic acid, whether LAA or DHAA, significantly reduced *M. phaseolina*-induced lesion development in ascorbic acid-pre-treated seedling cut-stems. The role of ascorbic acid in plant tolerance to pathogen-induced stress has been elucidated in transgenic potato plants with enhanced ascorbic acid levels when inoculated with *Phytophthora infestans* [50]. Their study showed that transgenic potato lines with enhanced ascorbic acid significantly reduced late blight lesions, H_2_O_2_, and malondialdehyde levels, a biomarker for oxidative stress. These authors also found that increased cellular ascorbic acid in transgenic plants aided in balancing cellular antioxidant levels, PR (pathogenesis-related) gene expression, and defense-associated hormones such as gibberellic acid and abscisic acid, which reduced senescence caused by *P. infestans*. Further, a previous study demonstrated ascorbic acid’s potential to defend against environmentally induced oxidative stress [36]. Our study that has shown the efficacy of exogenously applied ascorbic acid in scavenging H_2_O_2_ generated after inoculation by the necrotrophic fungus, *M. phaseolina*.

Pathogens with necrotrophic phases take advantage of induced, defense-associated ROS bursts and associated cell death for nutrition and concurrent colonization of senescent and necrotic tissue [51]. This phenomenon was observed in the soybean seedling cut-stem in-planta assay, where inoculated plants showed increased senescence as induced by *M. phaseolina.*

Tolerance to environmental stressors in plants such as salt stress is related to ROS scavenging. *Arabidopsis* mutant *vst1* showed increased tolerance to salt stress due to a ROS scavenging mechanism [52]. ROS detoxification or scavenging was performed by several antioxidants such as ascorbic acid, glutathione, thioredoxin, carotenoids, and other ROS scavenging enzymes, including SOD, GPx, and CAT [36]. Exogenous foliar application of ascorbic acid at 200 mg L^−1^ significantly improved the growth of flax cultivars under salt stress conditions [53]. A study by Shao et al. [54] demonstrated that ascorbic acid protects metabolic processes against H_2_O_2_ and minimizes H_2_O_2_-associated oxidative damage [54]. Our result is also consistent with this finding, as the exogenous application of ascorbic acid showed reduced senescence induced by *M. phaseolina*. As measured by AUDPC, disease progression was significantly slowed when either LAA or DHAA was applied to cut stems, suggesting the phenotype prediction of ROS scavenging by ascorbic acid. Additionally, the ROS quantification assay suggested the increased concentration of ROS generation as induced by *M. phaseolina* demonstrated the oxidative stress impact in plants. Ascorbic acid pre-treatment demonstrated a significantly lower level of H_2_O_2_ content, supporting a ROS quenching mechanism of ascorbic acid.

Charcoal rot is a plant disease that manifests during high temperature and water-limiting conditions. These environmental stressors contribute to pre-mature plant senescence, a situation that may be exploited by the necrotrophic phase of *M. phaseolina*. Furthermore, combinations of stressors are expected to exacerbate plant productivity in the future as they do now [55]. Therefore, understanding potential mechanisms by which charcoal rot, and other stress-associated diseases, may be mitigated, including quenching ROS-mediated responses, is an area of continued research interest.

## Figures and Tables

**Table 1:** Pre-treatments and treatments used in the soybean seedling stem necrosis assay.

Pre-Treatment (Day −1)	Treatment(s) (Day +0)
No pre-treatment	Agar plug Inoculate w/MP ^1^ Hydrogen peroxide (H_2_O_2_)
Agar plug	L-ascorbic acid (LAA; reduced) Dehydroascorbic acid (DHAA; oxidized)
Inoculate w/MP	LAA DHAA
LAA	Agar plug Inoculate w/MP
DHAA	Agar plug Inoculate w/MP
H_2_O_2_	Inoculate w/MP

^1^ MP = *Macrophomina phaseolina*.

**Table 2:** ANOVA results for three isolates of *M. phaseolina* grown on L-ascorbic acid at 1 to 5 days post-plating (D1 to D5).

Source	DF		D1	D2	D3	D4	D5
Model	31	*F*	37.89	62.52	160.95	172.64	34.51
		*P*	<0.0001	<0.0001	<0.0001	<0.0001	<0.0001
Replication	2	*F*	1.16	0.96	1.59	3.58	0.21
		*P*	0.3208	0.3891	0.2128	0.0341	0.8073
Isolate (I)	2	*F*	5.23	7.25	25.77	37.01	114.24
		*P*	0.0082	0.0015	<0.0001	<0.0001	<0.0001
Concentration (C)	9	*F*	120.26	210.35	542.98	580.33	114.24
		*P*	<0.0001	<0.0001	<0.0001	<0.0001	<0.0001
I *x* C	18	*F*	4.42	1.59	2.66	2.64	1.30
		*P*	<0.0001	0.0933	0.0025	0.0027	0.2244

**Table 3:** ANOVA results for H_2_O_2_ concentrations (mM) in soybean stem tissue after selected treatments with L-ascorbic acid.

Source	DF ^1^	SS	MS	*F*	*P*
Model	5	20,299.731	4059.946	17.00	0.0017
Replication	2	426.162	213.081	0.89	0.4580
Treatment	3	19,873.569	6624.523	27.73	0.0006
Error	6	1433.281	238.880		
Total	11	21,733.012			

^1^ DF = degrees of freedom, SS = sum of squares, MS = mean squares, *F* = *F*-statistic, *P* = *P*-value

## Data Availability

The data found in this paper is available in a public repository. Please see: https://doi.org/10.6084/m9.figshare.21207656.v1, https://doi.org/10.6084/m9.figshare.21207677. v1, and https://doi.org/10.6084/m9.figshare.21207707.v1 (accessed on 25 May 2022).

## References

[1] Hartman G.L., Rupe J.C., Sikora E.J., Domier L.L., Davis J.A., Steffey K.L. (2015). Compendium of Soybean Diseases and Pests.

[2] Živanov S.T., Dedić B.D., Dimitrijević A., Duśanić N., Jocić S., Miklič V., Kovačević B., Miladinović D. (2019). Analysis of genetic diversity among *Macrophomina phaseolina* (Tassi) Goid. Isolates from Euro-Asian countries. J. Plant Dis. Protect..

[3] Basandrai A.K., Pandey A.K., Somta P., Basandrai D. (2021). *Macrophomina phaseolina*-host interface: Insights into an emerging dry root rot pathogen of mungbean and urdbean, and its mitigation strategies. Plant Pathol..

[4] Rogers L.W., Koehler A.M. (2021). Nondestructive sampling to monitor *Macrophomina phaseolina* root colonization in overwintering *Stevia*. Plant Health Progr..

[5] Masi M., Sautua F., Zatout R., Castaldi S., Arrico L., Isticato R., Pescitelli G., Carmona M.A., Evidente A. (2021). Phaseocyclopentenones A and B, phytotoxic penta-and tetrasubstituted cyclopentenones produced by *Macrophomina phaseolina*, the causal agent of charcoal rot of soybean in Argentina. J. Nat. Prod..

[6] Bowers G.R., Russin J.S., Heatherly L.G., Hodges H.F., Heatherly L.G., Hodges H.F. (1999). Soybean disease management. Soybean Production in the Midsouth.

[7] Islam M.S., Haque M.S., Islam M.M., Emdad E.M., Halim A., Hossen Q.M., Hossain M.Z., Ahmed B., Rahim S., Rahman M.S. (2012). Tools to kill: Genome of one of the most destructive plant pathogenic fungi *Macrophomina phaseolina*. BMC Genom..

[8] Twizeyimana M., Hill C.B., Pawlowski M., Paul C., Hartman G.L. (2012). A cut-stem inoculation technique to evaluate soybean for resistance to *Macrophomina phaseolina*. Plant Dis..

[9] Doubledee M.D., Rupe J.C., Rothrock C.S., Bajwa S.G. (2018). Effect of root infection by *Macrophomina phaseolina* on stomatal conductance, canopy temperature and yield of soybean. Can. J. Plant Pathol..

[10] Hemmati P., Zafari D., Mahmoodi S.B., Hashemi M., Gholamhoseini M., Dolatabadian A., Ataei R. (2018). Histopathology of charcoal rot disease (*Macrophomina phaseolina*) in resistant and susceptible cultivars of soybean. Rhizosphere.

[11] Marquez N., Giachero M.L., Declerck S., Ducasse D.A. (2021). *Macrophomina phaseolina*: General characteristics of pathogenicity and methods of control. Front. Plant Sci..

[12] Bandara Y.M.A.Y., Weerasooriya D.K., Liu S., Little C.R. (2018). The necrotrophic fungus *Macrophomina phaseolina* promotes charcoal rot susceptibility in grain sorghum through induced host cell wall-degrading enzymes. Phytopathology.

[13] Boller T., Felix G. (2009). A renaissance of elicitors: Perception of microbe-associated molecular patterns and danger signals by pattern-recognition receptors. Ann. Rev. Plant Biol..

[14] Chisholm S.T., Coaker G., Day B., Staskawicz B.J. (2006). Host-microbe interactions: Shaping the evolution of the plant immune response. Cell.

[15] Jones J.D., Dangl J.L. (2006). The plant immune system. Nature.

[16] Millet Y.A., Danna C.H., Clay N.K., Songnuan W., Simon M.D., Werck-Reichhart D., Ausubel F.M. (2010). Innate immune responses activated in *Arabidopsis* roots by microbe-associated molecular patterns. Plant Cell.

[17] Faris J.D., Zhang Z., Lu H., Lu S., Reddy L., Cloutier S., Fellers J.P., Meinhardt S.W., Rasmussen J.B., Xu S.S. (2010). A unique wheat disease resistance-like gene governs effector-triggered susceptibility to necrotrophic pathogens. Proc. Natl. Acad. Sci. USA.

[18] Foley R.C., Kidd B.N., Hane J.K., Anderson J.P., Singh K.B. (2016). Reactive oxygen species play a role in the infection of the necrotrophic fungi, *Rhizoctonia solani* in wheat. PLoS ONE.

[19] Akram N.A., Shafiq F., Ashraf M. (2017). Ascorbic acid—A potential oxidant scavenger and its role in plant development and abiotic stress tolerance. Front. Plant Sci..

[20] Sarkar T.S., Biswas P., Ghosh S.K., Ghosh S. (2014). Nitric oxide production by necrotrophic pathogen *Macrophomina phaseolina* and the host plant in charcoal rot disease of jute: Complexity of the interplay between necrotrophy-host plant interactions. PLoS ONE.

[21] Turkan I. (2018). ROS and RNS: Key signaling molecules in plants. J. Exp. Bot..

[22] Hossain M.A., Bhattacharjee S., Armin S.M., Qian P., Xin W., Li H.Y., Burritt D.J., Fujita M., Tran L.S.P. (2015). Hydrogen peroxide priming modulates abiotic oxidative stress tolerance: Insights from ROS detoxification and scavenging. Front. Plant Sci..

[23] Černý M., Habánová H., Berka M., Luklová M., Brzobohatý B. (2018). Hydrogen peroxide: Its role in plant biology and crosstalk with signaling networks. Int. J. Mol. Sci..

[24] Habibi G., Ahmad P. (2014). Hydrogen peroxide (H_2_O_2_) generation, scavenging and signaling in plants. Oxidative Damage to Plants: Antioxidant Networks and Signaling.

[25] Nath M., Bhatt D., Prasad R., Gill S.S., Anjum N.A., Tuteja N. (2016). Reactive oxygen species generation-scavenging and signaling during plant-arbuscular mycorrhizal and *Piriformospora indica* interaction under stress condition. Front. Plant Sci..

[26] Caverzan A., Piasecki C., Chavarria G., Stewart C.N., Vargas L. (2019). Defenses against ROS in crops and weeds: The effects of interference and herbicides. Int. J. Mol. Sci..

[27] Saleh A.A., Ahmed H.U., Todd T.C., Travers S.E., Zeller K., Leslie J.F., Garett K.A. (2010). Relatedness of *Macrophomina phaseolina* isolates from tallgrass prairie, maize, soybean and sorghum. Mol. Ecol..

[28] Babu B.K., Saxena A.K., Srivastava A.K., Arora D.K. (2007). Identification and detection of *Macrophomina phaseolina* by using species-specific oligonucleotide primers and probe. Mycologia.

[29] AAT Bioquest, Inc (2021). Quest Graph^TM^. IC_50_ Calculator. https://www.aatbio.com/tools/ic50-calculator.

[30] Veljovic-Jovanovic S., Noctor G., Foyer C.H. (2002). Are leaf hydrogen peroxide concentrations commonly overestimated? The potential influence of artefactual interference by tissue phenolics and ascorbate. Plant Physiol. Biochem..

[31] Liu Y.H., Offler C.E., Ruan Y.L. (2014). A simple, rapid, and reliable protocol to localize hydrogen peroxide in large plant organs by DAB-mediated tissue printing. Front. Plant Sci..

[32] Cheeseman J.M. (2006). Hydrogen peroxide concentrations in leaves under natural conditions. J. Exp. Bot..

[33] Krzywinski M., Altman N. (2014). Visualizing samples with box plots. Nat. Meth..

[34] Botanga C.J., Bethke G., Chen Z., Galillie D.R., Fiehn O., Glazebrook J. (2012). Metabolite profiling of *Arabidopsis* inoculated with *Alternaria brassicicola* reveals that ascorbate reduces disease severity. Mol. Plant Micr. Interact..

[35] Miguel G., Fontes C., Martins D., Neves A., Antunes D. (2006). Effects of post-harvest treatment and storage time on the organic acid content in Assaria and Mollar pomegranate (*Punica grantum* L.) fruit. Ital. J. Food Sci..

[36] Khan A., Iqbal I., Shah A., Nawaz H., Ahmad F., Ibrahim M. (2010). Alleviation of adverse effects of salt stress in brassica (*Brassica campestris*) by pre-sowing seed treatment with ascorbic acid. J. Agric. Environ. Sci..

[37] Naz H.I.R.A., Akram N.A., Ashraf M. (2016). Impact of ascorbic acid on growth and some physiological attributes of cucumber (*Cucumis sativus*) plants under water-deficit conditions. Pak. J. Bot..

[38] Noctor G., Foyer C.H. (1998). Ascorbate and glutathione: Keeping active oxygen under control. Ann. Rev. Plant Biol..

[39] Ye N., Zhu G., Liu Y., Zhang A., Li Y., Liu R., Shi L., Jia L., Zhang J. (2012). Ascorbic acid and reactive oxygen species are involved in the inhibition of seed germination by abscisic acid in rice seeds. Journal Exp. Bot..

[40] Khan A., Ashraf M. (2008). Exogenously applied ascorbic acid alleviates salt-induced oxidative stress in wheat. Environ. Exp. Bot..

[41] Noctor G., Mhamdi A., Foyer C.H. (2014). The roles of reactive oxygen metabolism in drought: Not so cut and dried. Plant Physiol..

[42] Mittler R., Vanderauwera S., Gollery M., Van Breusegem F. (2004). Reactive oxygen gene network of plants. Trends Plant Sci..

[43] Van Doorn W.G., Ketsa S. (2014). Cross reactivity between ascorbate peroxidase and phenol (guaiacol) peroxidase. Postharv. Biol. Technol..

[44] Amin B., Mahleghah G., Mahmood H.M.R., Hossein M. (2009). Evaluation of interaction effect of drought stress with ascorbate and salicylic acid on some of physiological and biochemical parameters in okra (*Hibiscus esculentus* L.). Res. J. Biol. Sci..

[45] Chowdhury S., Basu A., Kundu S. (2017). Biotrophy-necrotrophy switch in pathogen evoke differential response in resistant and susceptible sesame involving multiple signaling pathways at different phases. Sci. Rep..

[46] Boguszewska D., Zagdańska B. (2012). ROS as signaling molecules and enzymes of plant response to unfavorable environmental conditions. Oxidative Stress–Molecular Mechanisms and Biological Effects.

[47] Nandini Y., Samir S. (2016). Reactive oxygen species, oxidative stress and ROS scavenging system in plants. J. Chem. Pharm. Res..

[48] Maurino V.G., Flügge U.-I. (2008). Experimental systems to assess the effects of reactive oxygen species in plant tissues. Plant Signal. Behav..

[49] Gill S.S., Singh L.P., Gill R., Tuteja N., Tuteja N., Gill S.S., Tiburcio A.F., Tuteja R. (2013). Generation and scavenging of reactive oxygen species in plants under stress. Improving Crop Resistance to Abiotic Stress.

[50] Chung I.M., Venkidasamy B., Upadhyaya C.P., Packiaraj G., Rajakumar G., Thiruvengadam M. (2019). Alleviation of *Phytophthora infestans* mediated necrotic stress in the transgenic potato (*Solanum tuberosum* L.) with enhanced ascorbic acid accumulation. Plants.

[51] Govrin E.M., Levine A. (2000). The hypersensitive response facilitates plant infection by the necrotrophic pathogen *Botrytis cinerea*. Curr. Biol..

[52] Tsugane K., Kobayashi K., Niwa Y., Ohba Y., Wada K., Kobayashi H. (1999). A recessive *Arabidopsis* mutant that grows photoautotrophically under salt stress shows enhanced active oxygen detoxification. Plant Cell.

[53] El Hariri D.M., Sadak M.S., El-Bassiouny H.M.S. (2010). Response of flax cultivars to ascorbic acid and α-tocophorol under salinity stress conditions. Int. J. Acad. Res..

[54] Shao H.B., Chu L.Y., Lu Z.H., Kang C.M. (2008). Primary antioxidant free radical scavenging and redox signaling pathways in higher plant cells. Int. J. Biol. Sci..

[55] Rivero R.M., Mittler R., Blumwald E., Zandalinas S. (2021). Developing climate-resilient crops: Improving plant tolerance to stress combination. Plant J..

